# Inhibition of Monkeypox virus replication by RNA interference

**DOI:** 10.1186/1743-422X-6-188

**Published:** 2009-11-04

**Authors:** Abdulnaser Alkhalil, Sarah Strand, Eric Mucker, John W Huggins, Peter B Jahrling, Sofi M Ibrahim

**Affiliations:** 1 United States Army Medical Research Institute of Infectious Diseases, Fort Detrick MD, 21702, USA; 2 Emerging Viral Pathogens Section, National Institute of Allergy and Infectious Diseases, National Institutes of Health, Bethesda MD, 20894, USA

## Abstract

The *Orthopoxvirus *genus of *Poxviridae *family is comprised of several human pathogens, including cowpox (CPXV), *Vaccinia *(VACV), monkeypox (MPV) and *Variola *(VARV) viruses. Species of this virus genus cause human diseases with various severities and outcome ranging from mild conditions to death in fulminating cases. Currently, vaccination is the only protective measure against infection with these viruses and no licensed antiviral drug therapy is available. In this study, we investigated the potential of RNA interference pathway (RNAi) as a therapeutic approach for orthopox virus infections using MPV as a model. Based on genome-wide expression studies and bioinformatic analysis, we selected 12 viral genes and targeted them by small interference RNA (siRNA). Forty-eight siRNA constructs were developed and evaluated *in vitro *for their ability to inhibit viral replication. Two genes, each targeted with four different siRNA constructs in one pool, were limiting to viral replication. Seven siRNA constructs from these two pools, targeting either an essential gene for viral replication (A6R) or an important gene in viral entry (E8L), inhibited viral replication in cell culture by 65-95% with no apparent cytotoxicity. Further analysis with wild-type and recombinant MPV expressing green fluorescence protein demonstrated that one of these constructs, siA6-a, was the most potent and inhibited viral replication for up to 7 days at a concentration of 10 nM. These results emphasis the essential role of A6R gene in viral replication, and demonstrate the potential of RNAi as a therapeutic approach for developing oligonucleotide-based drug therapy for MPV and other orthopox viruses.

## Background

Monkeypox virus (MPV) was first identified in laboratory-maintained cynomolgus monkeys [1]. The virus is believed to have been circulating for a long time in numerous animal hosts, including squirrels, in central and western Africa. Early human infections with MPV, which was recognized in Zaire and later in Liberia and Sierra Leone, occurred through direct contact with infected animals [2]. However, person-to-person transmission was reported more recently [3]. Monkeypox disease manifestation is similar to that of smallpox, but with lower case fatalities and more localized pustular rash distribution [4]. Because vaccination against smallpox ceased in early 1980s after the disease was declared eradicated [5], current public immunity towards poxviruses is deemed non-protective and younger generations are considered completely immune naive. Thus, a surprising natural, incidental or deliberate release of virulent monkeypox or other orthopox viruses poses a serious threat to public health. Currently, there are no licensed drugs to treat poxvirus infections, and use of antiviral Cidofovir and ST-246 [6] may gradually erode with emergence of resistant viral strains [7,8] or further identification of limiting drug side effects [6,9]. Therefore, the need for new effective drugs and novel therapeutic strategies that can withstand field application challenges is paramount.

RNA interference (RNAi) is a natural mechanism for gene expression regulation and protection against insertion of foreign RNA in plant and mammalian cells [10]. RNAi-based studies have been particularly valuable in elucidating gene functions in a variety of prokaryotic and eukaryotic organisms [11]. Recent advances in siRNA delivery systems and selective targeting methodology leveraged the prodigious utility of the RNAi pathway as a therapeutic approach for infectious, neurodegenerative, cancer, and hereditary diseases [12].

The use of RNAi pathway as a new approach in antiviral drug discovery is particularly promising because viruses have relatively small genomes with a limited number of targetable genes. Furthermore, genetic distance between mammalian and viral genomes represents an advantage in minimizing off-target hits and reducing possible side effects [13]. Recent studies utilized RNAi to silence specific viral genes and identify its function [14], or to inhibit viral replication [15]. In this study, we developed siRNAs to target several monkeypox viral proteins, and demonstrated the application of this approach in identifying new drug targets and inhibiting viral replication in cell culture.

## Results

### Selecting MPV genes targets and screening siRNA

Monkeypox virus genome consists of 196,858-base pairs (bp) with 190 open reading frames of 60 amino acid residues or more [16]. Like other orthopox viruses, the MPV genome encodes for a number of enzymes and factors that are necessary for entry, self-replication, and maturation. The central region of the genome contains highly conserved genes that are essential for viral replication, and terminal regions contain less conserved genes that are important for virus-host interactions. In designing siRNA molecules, we selected 12 gene targets based on their temporal expression and functional significance, e.g., attachment, replication, and host immune modulation (Table 1). These targets varied in size from 132 to 3021 bp, and mapped to the region between 22056 and 114223 on MPV genome covering most of the conserved region.

**Table 1:** Selected siRNA Targets

**MPV ORF**	**VACV ORF**	**Gene size(bp)**	**Region**	**Known or predicted function**
A5L	A4L	846	113340-114185	39 kDa immunodominant virion core protein needed for the progression of IV to infectious IMV

A6R	A5R	486	114223-114708	Precursor of RNA polymerase 22 kDa

C14L	F8L	195	35828-36022	No information available

C2L	K2L	1128	26384-27511	Serine protease inhibitor-like, SPI-3; inhibition of the ability of infected cells to fuse

C3L	K3L	132	27672-27803	Interferon resistance; host defense modulator

E8L	D8L	915	103116-104030	IMV cell attachment, putative; blockage causes plaque reduction

F8L	E9L	3021	53691-56711	DNA polymerase, catalytic subunit

H1L	H1L	516	87256-87771	Tyrosine/serine protein phosphatase; blocks IFN-γ

H3L	H3L	975	88358-89332	IMV cell attachment; heparin binding surface protein involved in IMV maturation

I3L	I3L	810	61085-61894	Virosomal ssDNA-binding phosphoprotein; interacts with R2 subunit of ribonucleotide reductase

L5L	J5L	402	82891-83292	Essential for virus multiplication

P1L	N1L	354	22056-22409	Virokine; host defense modulator

Selected MPV gene targets for siRNA development with gene size, location across MPV genome, and known or predicted function of its orthologs in VACV.

Custom SMARTpool software (Dharmacon) was selected to design four siRNA sequences for each of the 12 selected MPV genes. Constructs of 19-21 nucleic acids were chemically synthesized and pooled at comparable concentrations to maximize efficacy. Screening was performed in rhesus macaque kidney epithelial cells (LLC-MK2). This cell line was selected based on experimental transfection efficiency, host relevance, and ability to support replication of used MPV isolate.

**Figure 1:**
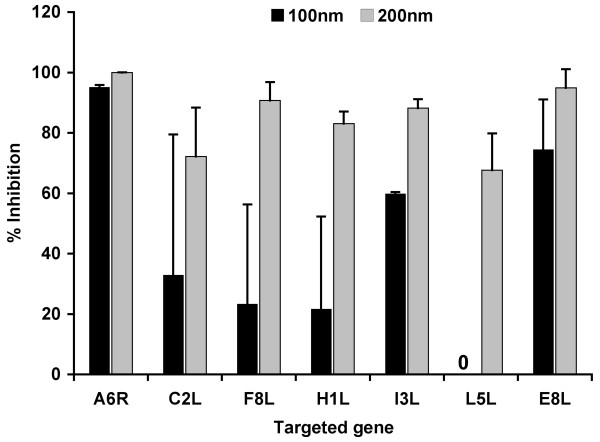
**Representative results from screened siRNA pools designed to specifically target selected MPV genes**. MK2 cells were transfected with a set of four multiplexed siRNA constructs targeting a single gene at a time. Parallel control cells were transfected with scrambled siRNAs under identical conditions to ensure antiviral specificity. Preliminary siRNA pools evaluation was carried at concentrations of 100 and 200 nM. Transfected cells were infected with MPV, and viral replication was assayed after incubation for 48 h by plaque assay. Presented results are relative to viral replication in mock-transfected cells ± one standard deviation. Multiplexed siRNA pools targeting A6R and E8L genes, i.e., siA6 and siE8, exhibited a strong dose-dependent antiviral effect with no or negligible toxicity at both concentrations.

For a preliminary evaluation of antiviral properties of the 12 siRNA pools, cultured LLC-MK2 cells in 24 wells plate were transfected with each of the multiplexed siRNA pools at 100 or 200 nM overnight. Cells were then infected with 100 pfu/well of MPV-KK. Infected cells were incubated for 48 h post infection (PI) at which time the cells were harvested and viral titer was measured by using the plaque assay. Percentage of viral replication rates in siRNA-transfected cells relative to replication in mock-transfected cells are shown in Fig 1. Inhibition of viral replication varied significantly based on targeted gene and used siRNA concentrations. For example, while anti-L5 siRNA pool showed little or no effect on viral replication at 100 nM, pools targeting A6R gene (siA6) or E8L gene (siE8) exhibited 95% and 78% at 100 nM respectively (Fig. 1). Differences in inhibition potency of siRNA polls at high 200 nM concentration were less pronounced generally, and most pools showed more than 50% inhibition.

Because transfection with siRNAs can be cytotoxic, especially at high concentrations [17], and consequently will affect viral replication [18], we examined the morphology of transfected cells using phase-contrast light microscopy and followed emergence of any cytopathic signs such as changes in the refractive index or cytoplasmic shrinkage of the cells. Alternatively we used vital dyes, such as Trypan blue. No signs of cytotoxicity were observed in cells treated with all tested siRNA pools at 100 nM, and only three out of the 12 siRNA pools induced 15-35% decrease in cells viability after 72 h of transfection at concentration of 200 nM (data not shown).

### Identifying the most potent single siRNA construct from inhibitory siRNA pools and defining its IC_50_

**Figure 2:**
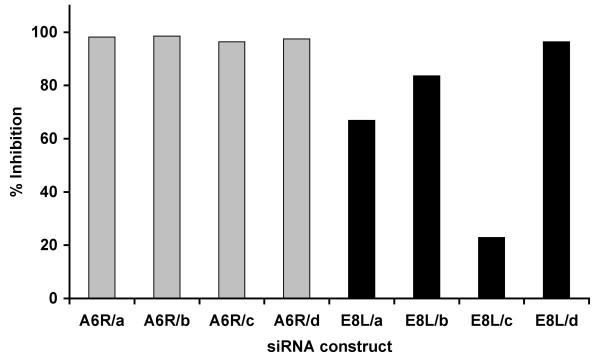
**Identifying the most potent antiviral siRNA construct within siA6 and siE8 pools**. MK2 cells transfected with 40 nM of one of the four constructs multiplexed in siA6 or siE8 pools, i.e., siA6R-a, -b, -c, -d or siE8L-a, -b, -c, -d. Transfected cells were infected with MPV and viral replication was determined at 48 h PI using plaque assay. Results are presented as a percentage of average plaque counts in treated cells to that in mock transfected control cells. siA6-a and siE8-d showed the strongest antiviral effect.

Each of the two siRNAs that exhibited the most significant inhibition of viral replication, siA6 and siE8, consisted of a pool of four different siRNA sequences that targeted various regions of the A6R and E8L genes (Table 2). Hence, we anticipated that each construct of the two pools will have different antiviral properties. To identify the most potent sequence within the two pools, we transfected LLC-MK2 cells with each of the siRNA sequences individually at concentration of 40 nM for 18 h. Transfected cells were infected with MPV-KK, and viral replication was evaluated 48 h PI. All four sequences of siA6 pool severely inhibited viral replication, with siA6-a and siA6-b being the most potent, achieving more than 95% inhibition. Interestingly, treating cells with the siA6 pool didn't result in appreciably stronger viral inhibition than treatment with the most potent siA6-a alone. The antiviral properties of siRNA sequences targeting E8L varied significantly and while siE8-d inhibited viral replication by more than 90%, siE8-c exhibited only a little more than 20% inhibition (Fig. 2).

**Table 2:** Sequences of siRNA constructs in the siRNA pools targeting A6R, and E8L genes.

**Targeted MPV gene**	**Used name**	**siRNA sequence**
E8L	siE8L-a	CGACAATAGTGTTCTGAAT
	siE8L-b	CGAATATCGTTGACTCATA
	siE8L-c	GCGCAGACATATTAGCGGA
	siE8L-d	GAATAGCGGTGAGTATAAA

A6R	siA6R-a	GAGAATTGTTGTCGGTAAA
	siA6R-b	TGAAATAGCGGGTATAATA
	siA6R-c	GCTCTTAAACGACGCTATA
	siA6R-d	GTCCTATAGTCATCGAAAA

Each sequence consists of 19 nucleotides.

Sequence of the most effective antiviral siRNA pools. Eight sequences each consists of 19 nucleotides with UU overhang were developed to target A6R, or E8L MPV genes. Sequences were BLAST searched against GenBank database to reduce potential for interference with host, off target effects, and optimize specificity.

**Figure 3:**
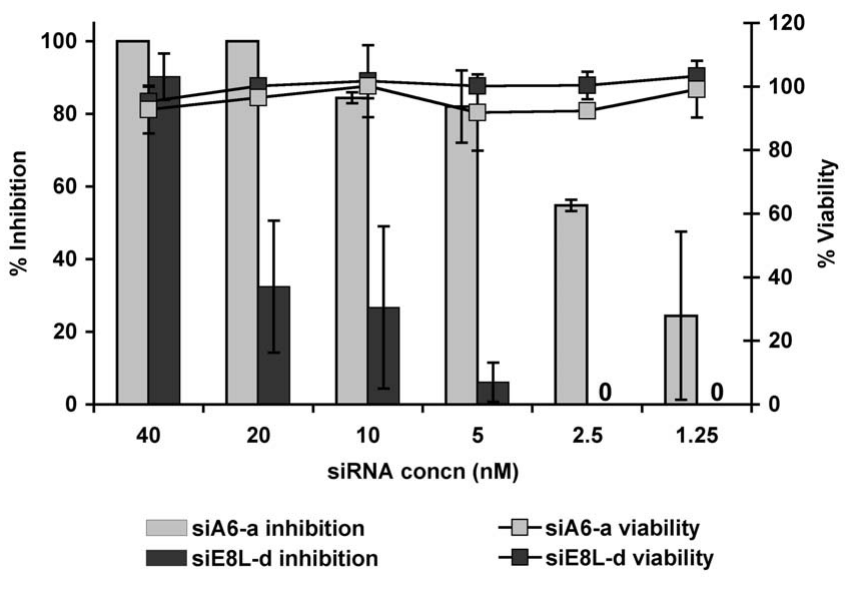
**Dose-response of siA6-a and siE8-d**. MK2 cells were transfected with either siA6-a or siE8-d at six different concentrations ranging between 40 to 1.25 nm. Transfected cells were infected with MPV and viral titer was identified at 48 h PI by plaque assay. Results (bars) are expressed as a ratio of the average plaque counts from treated wells to counts in parallel mock-transfected cells ± one standard deviation. Toxicity of both constructs were examined under identical conditions using (3-(4, 5-dimethylthiazolyl-2)-2, 5-diphenyltetrazolium bromide) in MTT assay. Results (lines) are normalized to viability of mock-transfected control cells (secondary Y-axis). Transfected cells maintained over 90% of their viability across tested range of siRNA concentrations, highlighting siRNA specificity and low toxicity.

To characterize the antiviral properties of most potent siRNA constructs, namely siA6-a and siE8-d, LLC-MK2 cells were transfected with one fold serial dilution of each construct to cover a range of 40 to 1.25 nM in six concentrations. Overnight transfected cells were infected with MPV at 100 pfu/well and viral replication was examined at 48 hours PI (Fig 3). siA6-a showed average viral replication inhibition of 23% at the lowest tested concentration of 1.25 nM, and complete inhibition at concentrations of 20 nM and higher. In agreement with previous assays, siE8-d maintained weaker antiviral activity than siA6-a and concentrations of ≤ 2.5 nM were inefficacious. The estimated *IC50 *for siA6-a would be less than 5 nM under the experimental conditions described above.

We used 3-(4,5-Dimethylthiazol-2-yl)-2,5-diphenyltetrazolium bromide (MTT) to measure the effect of siA6-a and siE8-d on cells viability. Transfected mock-infected cells that had been handled identically to virus infected cells maintained more than 90% viability at all siA6-a or siE8-d concentrations (Fig. 3).

### siA6-a exhibits a surprising in vitro half life

**Figure 4:**
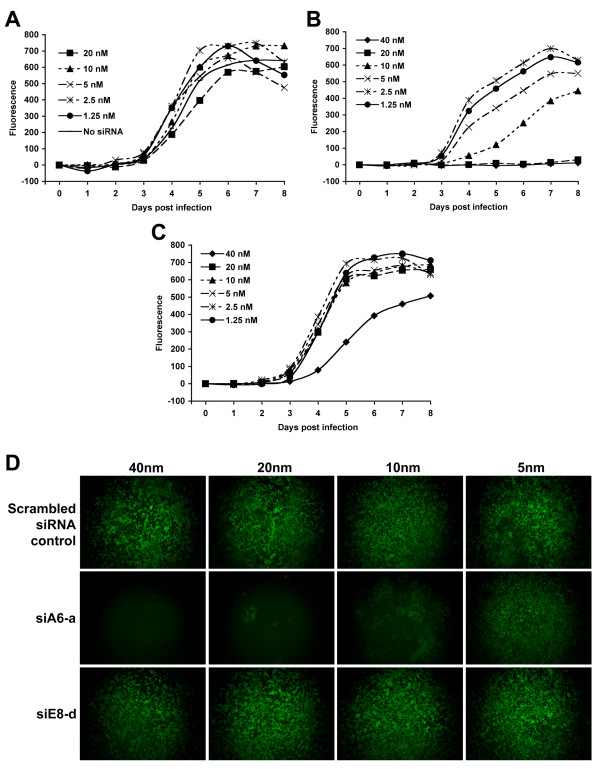
**Inhibition of MPV-GFP replication by siA6-a and siE8-d**. Cells transfected with various concentrations of scrambled non-targeting siRNA (A), siA6-a (B) or siE8-d (C) were infected with MPV-GFP, and viral replication was followed for 7 days PI by measuring the fluorescence increase in culture. (A) Cells treated with scrambled siRNA did not exhibit any antiviral activity and viral replication was similar to mock-transfected cells. (B) siA6-a maintained its potent antiviral activity up to 7 days PI in concentrations higher than 10 nm. (C) Inhibition by siE8-d was significant only at 40 nm with rapid degrading activity reflecting relative low stability. (D) Randomly picked images from virus infected-transfected cells at day 6 PI. Comparable fluorescence in cells transfected with scrambled siRNA at concentrations of 40 nm or less (upper panel). Cells transfected with siA6-a at concentrations of 10 nm or more did not show significant increase in fluorescence (middle panel), similar to those treated with 100 μM of Cidofovir (not shown) underscoring the potent antiviral function of siA6-a. Cells treated with siE8-d showed significant viral replication at concentrations of 20 nm or less as indicated by fluorescence intensity similarity with cells treated with scrambled non-inhibitory siRNA (last panel). Uninfected-transfected cells remained viable and no signs of cytopathy were detected until the end of the experiment (not shown).

We used MPV expressing green fluorescence protein (MPV-GFP) [19] to reproduce the results obtained using wild-type MPV-KK, examine the relationship of viral replication rate and virucidal properties of siA6-a or siE8-d, and to assess the stability of these two siRNA constructs over extended culture. Cells were transfected for 18 h with either siA6-a or siE8-d at the same concentration range described above. Transfected cells were infected with 2000 pfu/well of MPV-GFP, and viral replication was followed for 7 days PI by measuring fluorescence increase of the culture once every 24 h. Controls of untransfected-infected, transfected-uninfected, and non-targeting scrambled siRNA transfected-infected cells were included for results normalization and to confirmation of siRNA specificity. Consistent with plaque assay results obtained from wild-type MPV, the viral replication rates in cells transfected with scrambled non-targeting siRNA and in mock-transfected cells (Fig. 4A, D upper panel) were identical. No increase in fluorescence was observed in cells transfected with siA6-a at concentrations of 40 and 20 nm, which resembled the results observed in cells treated with 100 μM Cidofovir (not shown). Furthermore, viral replication rate was kept at less than half of its values in control cells as late as 7 days PI in cells treated with 10 nM of siA6-a, and only concentrations ≤ 5 nM exhibited mild or no viral inhibition (Fig. 4b, D middle panel). These results highlight the unusual stability of siA6-a construct and confirm its antiviral function regardless of virus replication rate. In contrary to our finding while using wild-type MPV, cells treated with siE8-d were more permissive to MPV-GFP replication and sharp increase in fluorescence was detected in cells treated at concentration ≤ 20 nM across all PI time points (Fig. 4C), and significant viral replication was observed at concentration of 40 nM (Fig, 4C, D lower panel). This variation in antiviral properties of siE8-d and dependence on experimental conditions was likely due to the known gradual decline of siRNA stability during extended incubation duration. The use of MPV-GFP required relatively long incubation period to reach the exponential phase of fluorescence amplification needed for adequate assessment of viral replication. Examination of transfected cells by phase contrast microscopy showed no cytopathic signs, underscoring the specific antiviral properties of siA6-a.

### Inhibition of MPV replication by siA6-a is MOI dependent

**Figure 5:**
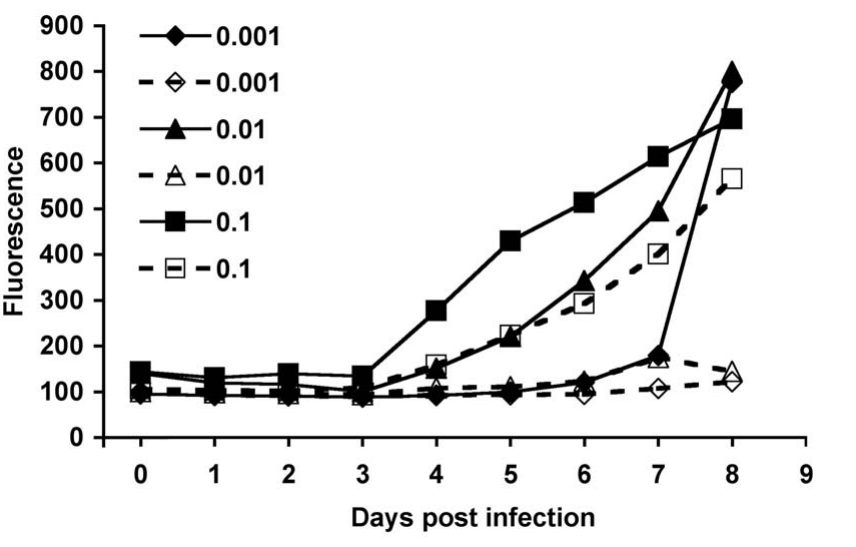
**MOI-dependent siA6-a inhibition of MPV replication**. Transfected MK2 cells with 20 nM of siA6-a were infected with MPV-GFP at MOIs of 0.001, 0.01, 0.1. Viral replication was traced by measuring the fluorescence increase in culture over duration of 7 days PI. Curves in dashed lines and empty markers represent siA6-a treated cells. Curves in solid lines and filled markers are mock transfected. siA6-a remained effective in cells infected with MPV at MOI of 0.01 for 8 days PI.

Successful siRNA application in the treatment of diseases depends on a variety of parameters, including stability of the siRNA, effective delivery, and efficacy against a high viral burden [20,21]. Stable and potent siRNA may have indications for post onset treatment as well as prophylaxis. We evaluated the antiviral properties of siA6-a through multiplicity of infection (MOI) range of 4 to 0.005. Our results showed that LLC-MK2 cells transfected with 20 nm of siA6-a remained refractory to viral replication after 8 days of MPV infection at an MOI of 0.01 or less (Fig. 5). Under identical conditions, siA6-a showed more than 50% inhibition of MPV-GFP replication at MOI of 0.1 in day 6 PI. Optimizing uptake and nuclease resistance properties of siA6-a, through chemical modifications that introduce lipophilic moieties and other chemical groups, would produce more potent derivatives that cover wider MOI range and last for longer durations [22].

## Discussion

Despite the outstanding success of vaccination in eradicating smallpox, the process was underlined with significant adverse reactions including inadvertent inoculation, ocular vaccinia, generalized vaccinia, eczema vaccinatum, progressive vaccinia, postvaccinial encephalopathy and encephalomyelitis, and fetal vaccinia. These complications can occur among primary vaccinated individuals or in secondary patients who are accidentally inoculated upon their contact with vaccinated individual [23,24]. Resorting to vaccine as a response measure to sudden poxvirus release is undermined further by increasing prevalence of immuno- compromised individuals and delayed immune protection inherent to vaccination. Broad use of vaccinia immune globulin (VIG) is hampered by its limited accessibility; hence, adhering to developing a chemical agent that is effective and safe for use in PI and chemoprophylaxis purposes presents a more favorable approach. While Cidofovir [25] and ST-246 [8] remain the drugs of choice for poxvirus infections, known side effects or propensity to develop resistant viral strains dictate cautious use [8,26,27].

In this study, we showed that RNAi can be used as a potent approach to reduce MPV replication in a sequence-specific manner. We screened 48 siRNA constructs in 12 pools targeting 12 monkeypox genes and examined their effect on viral replication. We showed that the siRNAs affected MPV replication in various degrees. Two siRNA pools exhibited substantial antiviral properties and reduced viral replication to less than 10% of its propagation in control untreated cells. A single siRNA construct targeting A6R gene suppressed viral replication to near completion with IC_50 _less than 10 nanomoles.

The observed disparity in siRNA efficacy among screened pools is consistent with a number of previous reports [28,29]. Although reasons for this variation are still not fully understood, it is accepted that delivery of siRNA in its optimal functional concentration to targeted cells, and other unpredictable sub-cellular events such as concentration of siRNA in endosomes or trapping in other vesicles are major causes of variation in siRNA efficacy studies [30]. Furthermore, larger forms of RNA undergo a number of sequential processing steps before they interact with RNAi machinery and achieve the intended biological function. This include processing of miRNA and shRNA by Dicer, assembly of resultant guide strand into the RNA-induced silencing complex (RISC), recognition of target viral RNA sequences and cleavage of targeted mRNA. Because these steps involve interactions of various secondary molecular conformations defined by diverse primary sequences they are likely to exhibit unequal efficiencies, which would factor into the observed variation in siRNAs potency. Thus, identifying an effective siRNA empirically remains a common tool in therapeutic applications of RNAi [31].

It is important to note that screened siRNA pools may have silenced the targeted genes, but without producing a phenotype or influencing MPV replication. This is essentially defined by the function of the targeted gene. Presence of viral or host compensating mechanisms for the function of knocked-down gene would obfuscate evaluation of siRNA efficacy further [32], especially when reduction or inhibition of replication is the endpoint in assessing siRNA potency. Alternatively, multiple genes with varied copy number [33] are found to contribute to the same phenotype or trait with different intensities. Using siRNA and reverse genetics to silence one or more of these genes and determine its function is intrinsically difficult. Similarly, genes associated with high translation turnover duration [34] or highly efficient protein synthesis mechanisms can sustain viral replication at low copy number. Nevertheless, our results provided reverse genetic evidence for the vitality of A6R and E8L in MPV replication, and further work is needed to clarify the knockdown of the other targets at the gene or protein level.

Poxvirus is the only known double-stranded DNA viral family that propagates in host cytoplasm and encodes most of the enzymes and factors necessary for transcription and replication of its material [35]. Once the virus enters into the host cytoplasm, it becomes uncoated to release its genetic information and component of early transcription system packaged within the core of the virion [36,37]. Many targeted RNA sequences will interact with viral and/or cellular proteins which would hamper, if not prevent, the ability of RNA-induced silencing complex to recognize its viral targets [38]. This may contribute to the variation we observed in the efficacy of single siRNA constructs within the same pool as in the case of siE8-d and siE8-c which target the same E8L gene.

The function of siA6-a and E8-d targets in MPV remain unidentified; however, their orthologs in vaccinia, A5R, and D8L, function as a precursor of RNA polymerase [39] and cell attachment protein [40]. Mutant vaccinia virus with dysfunctional A5 or D8 genes show no or severely perturbed viral replication. Soluble vaccinia D8 protein, which binds chondroitin sulfate (CS), interferes with adsorption of wild-type vaccinia and decreases viral propagation rate. The significant decrease of MPV replication in siE8-d treated cells, and the disruptive effect of soluble D8 on vaccinia adsorption with consequent lower rates of replication imply a sort of similarity in both gene functions and suggests possible role for CS in MPV cell entry. The presence of alternative MPV cell-attachment and adsorption mechanisms, such as binding of A27L protein with cell heparan sulfate HS described in vaccinia [41], may account for the incomplete replication inhibition of MPV replication despite knock-down of E8 gene.

Persistence of RNAi occurs for short period of time mainly due to the relatively short half life of siRNA and lack of an amplification mechanism in mammalian cells. The estimated 66 hours of RNAi persistence is relatively short due to siRNA hydrolysis and dilution over the course of cell division [42]. Recently D5R gene in vaccinia virus strain Western Reserve was introduced as a valid siRNA target *in vitro*[43]. Targeting this gene in vaccinia WR, CPV, and MPV led to 70% inhibition of viral replication at nanomolar levels of siRNA using different cell lines. The same work report an impressive prolonged prophylactic antiviral effect that lasted for 72 h at concentration of 100 nm. Surprisingly, our siA6-a maintained solid viral replication inhibition for more than 7 days PI in cell culture at concentration of 20 nm. This unusual stability may be due to a unique molecule tertiary structure and/or highly sensitive target. Further work is under way to access the pharmacokinetics of A6-a construct and address this point.

The antiviral effects of 20 nm of siA6-a and 100 nm of Cidofovir were comparable and seem to inhibit the replication of all virus forms. These two drugs target the virus directly by silencing gene expression or interfering with vDNA replication without perturbing host cell biology. However, easy synthesis and adjustment of siRNA sequence represent an extra advantage over other chemically synthesized drugs. This, in addition to recent advancements in sequencing capabilities and bioinformatics, enabled unprecedented flexibility to readapt siRNA molecules to function on any emerging resistant viral strains, enhance siRNA specificity, and reduce potential side effects. These tasks are made easier when the targeted microorganism and its host are genetically different. In our case, siA6-a didn't induce any signs of cytotoxicity and seemed not interfere with host cell biology even when used at concentrations up to ten times its IC_50_.

An alternative approach for developing antiviral drugs targets specific host functions necessary for viral replication. A good example of this class of targets is the epidermal growth factor receptor family of tyrosine kinases [44], which disrupt viral maturation and replication cycle when antagonized. A member of this family (ErbB-1) was inhibited by CI-1033, a drug that has been developed originally for its anticancer properties, and led to significant reduction in poxvirus activity [45]. However, CI-1033 seems to be more specific in targeting IMV and not EEV forms. This was evident from the described reduction in the size and not overall plaque count, and from the synergistic antiviral effect observed in cells co-treated with neutralizing virus antibodies and CI-1033. It remains unclear how inhibiting ErbB would affect host homeostasis. The complete inhibition of MPV replication by targeting A6R gene suggests that, unlike CI-1033, siA6-a acts on an indispensable viral function at a stage upstream to viral differentiation into distinct forms, and abolishes virus replication regardless of its form.

In conclusion, using RNAi pathway we identified two MPV genes that serve as potential drug targets during infection in cell culture. A6R and E8L genes of MPV were antagonized effectively using siRNA molecules, and constructs siA6-a and siE8-d disrupted MPV replication severely. siA6-a construct exhibited considerable stability and promising antiviral potency with IC_50 _less than 10 nm. Chemical modification study aiming to enhance A6-a stability and development of suitable siRNA delivery system are needed before assessing siA6-a in animal models.

## Materials and methods

### Viruses and cultures

Wild-type monkeypox virus, strain Katako Kombe (MPV-KK) and MPV expressing green fluorescent protein (MPV-GFP) were used to evaluate siRNA efficacy [19]. The viruses were propagated in Vero E6 cells maintained in Eagle's minimum essential medium with non-essential amino acids (EMEM/NEAA) supplemented with 2 mM L-glutamine, 10% of heat-inactivated fetal bovine serum (FBS), 10 mg/L Gentamycin, 250 μg/L Fungizone, and buffered with 10 mM HEPES [46]. Viral titers were determined by the plaque assay as described in [29]. Briefly, monolayers of confluent Vero-E6 cells in six-well plates were overlaid with 100-200 μl aliquots of serial dilutions of examined viral sample in triplicate. The virus was allowed to absorb for 1 h at 37°C with gentle mix for 30 sec each 15 min. Two to three ml of complete culture medium was added to each well, and plates were incubated until the development of clear plaques (≈ 4-5 days). Stock solution of 1.3 g/L of crystal violet, 30% formalin, and 5% ethanol was diluted with PBS (1:2) and used to fix and stain the cells. Plates were incubated 15-20 min at room temperature to allow clear staining. Cells were rinsed gently with PBS and plaques were counted on a light box.

For experiments involving MPV-GFP, plates were infected with 2000 plaque-forming units (pfu) per well unless otherwise indicated. Fluorescence readings were taken every 24 h by using a Gemini EM fluorescence reader and Softmax 4.7 software (Molecular Devices, Union City, CA). The results were normalized to average fluorescence in control cells. Data were expressed as means ± SD. Statistical analysis was performed using Student's t-test when appropriate.

### siRNA design, preparation, and transfection

Chemically synthesized siRNAs were custom-designed to target selected MPV genes by Dharmacon (Chicago, IL). siRNA sequences were BLAST-searched against the human genome database to assess possible cross-reactivity. For each targeted gene, four siRNAs were synthesized and pooled. Dried siRNA pools were reconstituted in rehydration buffer (100 mM KCl, 30 mM HEPES pH 7.5, 1 mM MgCl_2_) to a final concentration of 100 μM and stored at -80°C until used.

LLC-MK2 cells were used to evaluate the efficacy of the siRNAs. Confluent cells were briefly trypsinized, counted, and resuspended in culture medium at desired concentration. Cell suspension was used to seed 24-well culture plates (Costar, Lowell, MA), and plates were incubated for 24-48 h to allow multiplication of cells in antibiotic- and Fungizone-free medium before infection with virus. To transfect the cells, siRNA and the transfection reagent were complexed as recommended by the manufacturer (Dharmacon, Chicago, IL). Briefly, equal volume of 10× solution (relative to the final intended concentrations) of siRNA and transfection reagent were prepared and mixed together. The resultant 5× solutions mix was incubated for 30 min at room temperature to allow the formation of siRNA transfection complex. This was diluted to the intended final 1× concentration using OPTI-MEM-I (Invitrogen, Carlsbad, CA). Cells were washed twice gently with 0.5 ml of OPTI-MEM-I buffer and incubated with 1× transfection siRNA complex for 12 to 16 h prior to viral infection.

### Cells infection with virus

To infect siRNA transfected and non transfected control cells with MPV, incubation medium was replaced by the virus inoculum diluted to produce the desired MOIs using OPTI-MEM-I (GIBCO-Invitrogen, Carlsbad, CA). Cells were incubated with the virus seed for 30 min at room temperature to allow viral absorption, and gentle 15-sec shake every 10 min was done to ensure even viral distribution. Removed culture medium was added back, and cells were incubated in 93-95% relative humidity atmosphere at 37°C, 5% CO_2_.

### Toxicity assay

Cells viability was assessed using MTT Cell Proliferation assay (ATCC, Manassas, VA). Briefly, 10 μl of MTT (3-(4,5-Dimethylthiazol-2-yl)-2,5-diphenyltetrazolium bromide) was added to each well and the plates were incubated at 37°C, 5% CO_2 _until clear purple crystals precipitant appeared in cells cytoplasm (4-8 h). Cells were lysed by adding 100 μl of detergent (proprietary, ATCC kit) and incubated overnight or until the purple crystals were dissolved. Color intensity was measured by spectrophotometer (Tecan, Pittsburgh, PA) at 570 nm, and cytotoxicity was calculated as a ratio of absorbance in treated versus untreated cells.

## Competing interests

The authors declare that they have no competing interests.

## Authors' contributions

All authors read and approved this manuscript. AA was responsible for design and completion of the bulk of the research, as well as for data analysis and writing of this manuscript. SS carried out part of the experiments including plaque assays. EM contributed with critical thinking and planning towards experiments, carried out GFP-MPV based assays and maintained virus strains. JH and PJ were instrumental in providing support to the research in the form of both instruction and facilitating all virus culture aspects of the research, including establishing, selecting, and maintaining all virus strains. SMI was the Principal Investigator and is primarily responsible for all aspects of the funding, research design, interpretation, and writing of this manuscript.
